# Poly (I:C)-Induced microRNA-30b-5p Negatively Regulates the JAK/STAT Signaling Pathway to Mediate the Antiviral Immune Response in Silver Carp (*Hypophthalmichthys molitrix*) via Targeting CRFB5

**DOI:** 10.3390/ijms25115712

**Published:** 2024-05-24

**Authors:** Meng Liu, Huan Tang, Kun Gao, Xiqing Zhang, Zhenhua Ma, Yunna Jia, Zihan Yang, Muhammad Inam, Yunhang Gao, Guiqin Wang, Xiaofeng Shan

**Affiliations:** Department of Veterinary Medicine, College of Animal Science and Technology, Jilin Agricultural University, Changchun 130118, China; liumeng4610@163.com (M.L.); tanghuan202308@163.com (H.T.); kungao213@163.com (K.G.); zhangxiqing1020@163.com (X.Z.); mazhenhua1030@163.com (Z.M.); 15590074644@163.com (Y.J.); yyangzihan622@163.com (Z.Y.); dr.inam@sbbu.edu.pk (M.I.); sxf1997@163.com (X.S.)

**Keywords:** poly (I:C), silver carp, innate immunity, IFNs, miR-30b-5p, CRFB5

## Abstract

In aquaculture, viral diseases pose a significant threat and can lead to substantial economic losses. The primary defense against viral invasion is the innate immune system, with interferons (IFNs) playing a crucial role in mediating the immune response. With advancements in molecular biology, the role of non-coding RNA (ncRNA), particularly microRNAs (miRNAs), in gene expression has gained increasing attention. While the function of miRNAs in regulating the host immune response has been extensively studied, research on their immunomodulatory effects in teleost fish, including silver carp (*Hyphthalmichthys molitrix*), is limited. Therefore, this research aimed to investigate the immunomodulatory role of microRNA-30b-5p (miR-30b-5p) in the antiviral immune response of silver carp (*Hypophthalmichthys molitrix*) by targeting cytokine receptor family B5 (CRFB5) via the JAK/STAT signaling pathway. In this study, silver carp were stimulated with polyinosinic-polycytidylic acid (poly (I:C)), resulting in the identification of an up-regulated miRNA (miR-30b-5p). Through a dual luciferase assay, it was demonstrated that CRFB5, a receptor shared by fish type I interferon, is a novel target of miR-30b-5p. Furthermore, it was found that miR-30b-5p can suppress post-transcriptional CRFB5 expression. Importantly, this study revealed for the first time that miR-30b-5p negatively regulates the JAK/STAT signaling pathway, thereby mediating the antiviral immune response in silver carp by targeting CRFB5 and maintaining immune system stability. These findings not only contribute to the understanding of how miRNAs act as negative feedback regulators in teleost fish antiviral immunity but also suggest their potential therapeutic measures to prevent an excessive immune response.

## 1. Introduction

In vertebrates, the immune system has evolved to include two main types of immunity, innate and adaptive, which collectively protect the host against virus invasion [1]. Innate immunity serves as the initial defense against viral invasion. This system primarily operates by recognizing pathogen-associated molecular patterns (PAMPs) through pattern-recognition receptors (PRRs) of host cells, which subsequently initiate a series of signal pathways to regulate immune response [2]. The interferon (IFN) system is one of the innate immune mechanisms against viral infection. Upon stimulation by viruses or double-stranded RNA (dsRNA) analogs, type I IFNs bind to their receptor complexes, IFNAR1 and IFNAR2, leading to activation of the JAK/STAT signaling pathway. This activation induces the expression of interferon-stimulating genes (ISGs), which play a pivotal role in defending against viral invasion [3,4]. IFN-I in fish performs biological functions similar to those in mammals and is mainly categorized into group I IFN and group II IFN based on cysteine content. However, diverse receptor complexes comprising CRFB5/CRFB1 or CRFB5/CRFB2 are activated by group I and II IFNs. CRFB5 serves as a common receptor chain shared by all IFN-I, indicating its significant role in regulating the antiviral response to interferons in teleost fish [5]. While the characteristics and roles of CRFB5 have been extensively studied in teleost fish [6,7,8], the regulatory mechanism of CRFB5 mediated by non-coding genes has not been previously reported.

MicroRNAs (miRNAs) are approximately 21–23 nucleotides in length and represent a class of endogenous non-coding RNAs. They exert their regulatory effects by specifically binding to the 3’UTR of the target mRNAs, leading to mRNA degradation or the inhibition of translation. MiRNAs play crucial roles in regulating various physiological and pathological processes [9]. The regulatory role of miRNAs in the immune response was first discovered in 2004 [10], and subsequent studies elucidated their involvement in modulating the immune response by targeting immune-related genes. For instance, miR-15a-5p and miR-202-5p regulate antiviral responses by targeting TRIF and TRIM25, respectively [11,12]. Recently, extensive research has been conducted on the role of miRNA in mammals, with successive reports on the function of miRNA in immune modulation. However, investigations into the function of miRNA in teleost fish has gained increasing attention. Previous research has demonstrated the potential of miRNAs as regulatory factors in modulating the immunological response mediated by the JAK/STAT signaling pathway [13,14,15]. Nevertheless, there is a lack of research on the miRNA-mediated regulation of antiviral infection through the JAK/STAT signaling pathway specifically in teleost fish.

Silver carp (*Hypophthalmichthys molitrix*) is a filter-feeding freshwater fish widely distributed in major water bodies in China [16]. Silver carp have been a popular food source for Chinese citizens due to their richness in protein, vitamins, minerals, and amino acids. However, the increasing density of aquaculture, deterioration of water quality, and inadequacies in the seedling origin quarantine mechanism have led to the frequent occurrence of fish diseases, particularly viral infections, such as spring viraemia of carp virus (SVCV) [17], cyprinid herpesvirus 2 (CyHV-2) [18], and viral hemorrhagic septicemia virus (VHSV) [19], resulting in significant economic losses to the aquaculture.

Given the importance of controlling viral infections in silver carp for sustainable aquaculture development, this study investigated the expression of miR-30b-5p in response to poly (I:C) stimulation in silver carp. This study confirmed CRFB5 as one of the target genes of miR-30b-5p targets through a dual luciferase report system. Furthermore, this study elucidated the mechanism by which miR-30b-5p contributes to the antiviral response by regulating the JAK/STAT signaling pathway in silver carp through its targeting CRFB5. Overall, these results enrich the comprehension of the immunoregulatory mechanisms of miRNAs in silver carp and may offer new therapeutic targets for enhancing teleost fish resistance to viral diseases.

## 2. Results

### 2.1. Poly (I:C) Enhanced miR-30b-5p Expression

**Figure 1:**
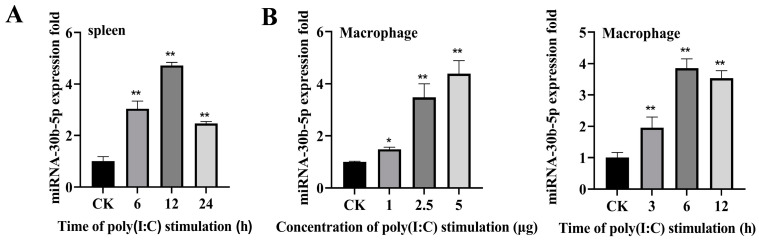
MiR-30b-5p expression in vivo and in vitro of silver carp following poly (I:C) stimulation. The spleen tissue (**A**) and the HKCs (**B**) had poly (I:C) treatment for different durations (6, 12, and 24 h) and were subsequently gathered to purify RNA, respectively. MiR-30b-5p expression was assessed using RT-qPCR. The internal control was provided by U6. Data are presented as the means ± SD (*n* = 3). Significance is shown as *, *p* < 0.05; **, *p* < 0.01 in comparison to the controls.

In order to investigate miRNAs involved in the silver carp’s antiviral defense mechanism, we examined the miRNA expression profile in silver carp spleen tissue following poly (I:C) stimulation. Deep sequencing analysis revealed a differential expression of several miRNAs, among which miR-30b-5p showed a significant increase upon poly (I:C) stimulation (Figure S1). The expression pattern of miR-30b-5p in vivo and in vitro was further verified by RT-qPCR. As shown in Figure 1A,B, the expression of miR-30b-5p was up-regulated in the poly (I:C)-stimulated spleen and HKCs compared with the control group CK. These findings suggest that miR-30b-5p may play a role in regulating the immune response triggered by poly (I:C).

### 2.2. MiR-30b-5p Inhibited the Production of Antiviral Genes

**Figure 2:**
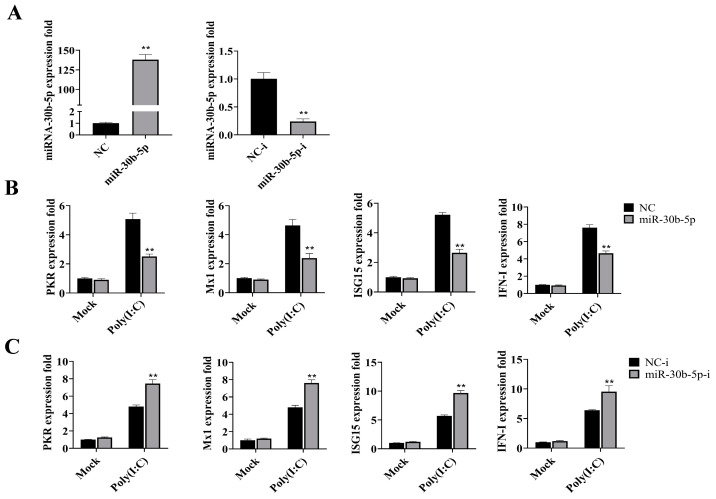
miR-30b-5p involvement in regulating the expression of antiviral genes in poly (I:C)-treated HKCs of silver carp. (**A**) Silver carp HKCs were transfected with miR-30b-5p or negative control (NC), as well as miR-30b-5p inhibitor (miR-30b-5p-i) or negative control inhibitor (NC-i), at a final concentration of 100 nM. At 48 h post transfection, miR-30b-5p expression was quantified by RT-qPCR and normalized to U6. (**B**,**C**) Silver carp HKCs were transfected with miR-30b-5p or NC (**B**) and miR-30b-5p-i or NC-i (**C**). After 48 h, cells were treated with poly (I:C) for 6 h, and the expression levels of *PKR*, *Mx1*, *ISG15*, and *IFN-I* were analyzed by RT-qPCR. Results were normalized to 1 in control cells. Data are presented as the means ± SD (*n* = 3). Significance is shown as **, *p* < 0.01 versus the controls.

The use of miRNA mimics is known to enhance the expression of mature miRNA in cells, while miRNA inhibitors have the opposite effect. To investigate the impact of miR-30b-5p on the antiviral immune response of silver carp, we transfected miRNA mimics (miR-30b-5p) and inhibitors (miR-30b-5p-i) into silver carp HKCs and examined their effects on the expression of antiviral genes. As depicted in Figure 2A, HKCs that were transfected with miR-30b-5p showed a 136-fold increase in their expression, whereas the miR-30b-5p-i transfection resulted in a reduction that was greater than 70%. Subsequent analysis revealed that miR-30b-5p overexpression markedly suppressed the expression of key antiviral genes, including Protein kinase R (*PKR*), myxovirus resistance protein 1 (*Mx1*), interferon stimulated gene 15 (*ISG15*), and type I interferon (*IFN-I*), following poly (I:C) stimulation (Figure 2B). Conversely, the inhibition of miR-30b-5p led to the up-regulation of these antiviral genes (Figure 2C). These results indicate that miR-30b-5p acts as a negative regulator of the antiviral immune response elicited by poly (I:C).

### 2.3. MiR-30b-5p Targets CRFB5

MiRanda (v3.3a) and Targetscan (v5.0) were the software employed to forecast the probable miR-30b-5p targets. As shown in Table S2, we predicted that miR-30b-5p has 10 target genes. CRFB5, a homolog of IFNAR1, plays a crucial role in the antiviral response mediated by IFN-I. Therefore, the target gene CRFB5 was chosen for verification.

**Figure 3:**
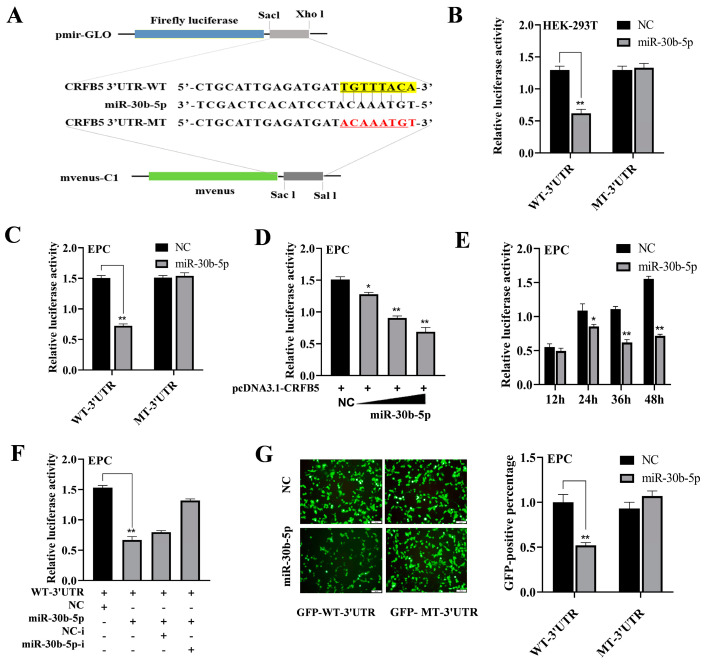
miR-30b-5p targets silver carp CRFB5. (**A**) Schematic diagram illustrating miR-30b-5p target site prediction in CRFB5 3′UTR and the process of constructing reporter plasmid; The yellow shadow region: the binding sites; The red characters: the mutation sites. (**B**,**C**) Co-transfection of HEK-293T cells (**B**) or EPC cells (**C**) was performed using either WT-3′UTR or MT-3′UTR reporter plasmids along with miR-30b-5p or NC, and luciferase activity was assessed 48 h post transfection. (**D**,**E**) Co-transfection of EPC cells was carried out with WT-3′UTR, along with miR-30b-5p or NC in a concentration (**D**) and time gradient (**E**) manner, and luciferase activity was assessed post transfection. (**F**) Luciferase activity was evaluated 48 h after the co-transfection of WT-3′UTR and miR-30b-5p with miR-30b-5p inhibitor (miR-30b-5p-i) or negative control inhibitor (NC-i) in EPC cells. (**G**) GFP-WT-3′UTR or GFP-MT-3′UTR was co-transfected with NC or miR-30b-5p in EPC cells; scale bars: 50 μm. Fluorescence intensity was measured 48 h post transfection. Data are presented as the means ± SD (*n* = 3). Significance is shown as *, *p* < 0.05; **, *p* < 0.01 in comparison to the controls.

To validate the interaction between miR-30b-5p and CRFB5, we constructed wild-type and mutant plasmids containing CRFB5-3′UTR sequences (Figure 3A). The co-transfection of miR-30b-5p with the wild-type plasmid resulted in a significant reduction in luciferase activity, while no change was observed with the mutant plasmid (Figure 3B,C). This effect was concentration- and time-dependent (Figure 3D,E) and could be reversed by co-transfection with miR-30b-5p-i (Figure 3F). Furthermore, the co-transfection of miR-30b-5p with the wild-type plasmid led to a down-regulation of GFP expression (Figure 3G), confirming silver carp’s CRFB5 as a direct target of miR-30b-5p, which has a target binding site in the 3′UTR of CRFB5.

### 2.4. MiR-30b-5p Suppresses the Expression of CRFB5 at the Post-transcriptional Level

**Figure 4:**
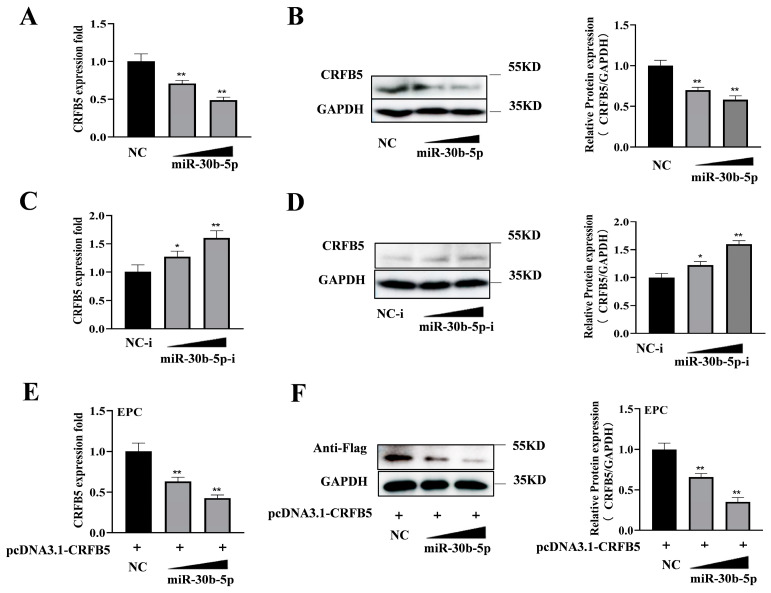
miR-30b-5p suppresses CRFB5 expression at the post-transcriptional level. (**A**,**C**) Silver carp HKCs were transfected with miR-30b-5p or negative control (NC) (**A**) and miR-30b-5p inhibitor (miR-30b-5p-i) or negative control inhibitor (NC-i) (**C**). RT-qPCR was used to assess the mRNA expression levels of CRFB5 48 h later and standardized to GAPDH. (**B**,**D**) Silver carp HKCs were transfected with miR-30b-5p or NC (**B**) and miR-30b-5p-i or NC-i (**D**). After 48 h, CRFB5 protein levels were determined by Western blotting. E and F, EPC cells were co-transfected with pCDNA3.1-CRFB5, containing the 3′UTR and the entire CDS region of CRFB5, along with miR-30b-5p or NC. mRNA (**E**) and protein (**F**) levels of CRFB5 were assessed by RT-qPCR and Western blotting, respectively, at 48 h post transfection. Data are presented as the means ± SD (*n* = 3). Significance is shown as *, *p* < 0.05; **, *p* < 0.01 in comparison to the controls.

MiRNAs may down-regulate target gene expression through mRNA degradation or the inhibition of translation, so miR-30b-5p was investigated for its role in regulating CRFB5 expression. As shown in Figure 4A,B, the transfection of miR-30b-5p into silver carp HKCs resulted in a dose-dependent reduction in CRFB5 mRNA and protein levels, whereas the transfection of miR-30b-5p inhibitors led to an increase in CRFB5 expression (Figure 4C,D). Furthermore, co-transfection of the CRFB5 full-length plasmid with miR-30b-5p into EPC cells also resulted in a reduced CRFB5 expression (Figure 4E,F). These findings suggest that miR-30b-5p directly targets and suppresses the post-transcriptional expression of CRFB5.

### 2.5. CRFB5 Is Involved in the Poly (I:C)-Induced Immune Response through the JAK/STAT Signaling Pathway

**Figure 5:**
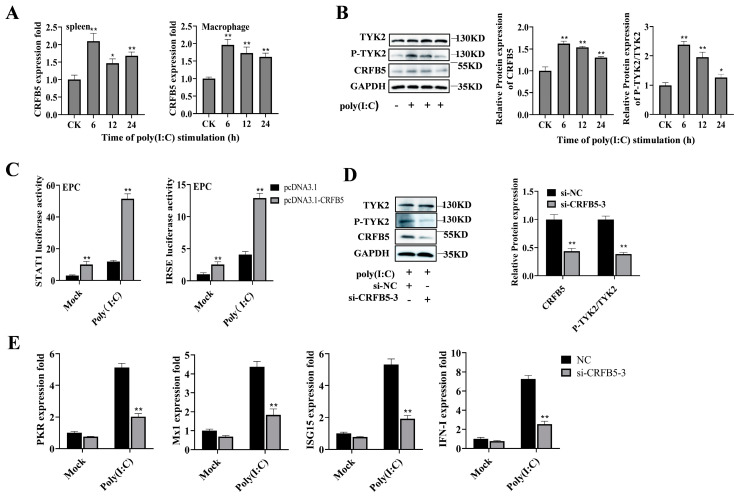
CRFB5 involvement in the poly (I:C)-induced immune response via the JAK/STAT signaling pathway. (**A**) Expression profile of *CRFB5* in silver carp spleen (**A**) and HKCs (**B**) stimulated by poly (I:C). (**B**) Poly (I:C) stimulated HKCs in a time gradient manner, and the expression levels of CRFB5 and phosphorylated P-TYK2 were detected by Western blotting. (**C**) After the co-transfection of EPC cells with the full-length expression plasmid pCDNA3.1-CRFB5 or the negative control pCDNA3.1, along with the IRSE luciferin reporter plasmid or STAT1 at 48 h, poly (I:C) was used to activate the cells for 6 h. Luciferase activity was measured. (**D**,**E**) Silver carp HKCs were transfected with si-CRFB5-3 or si-NC for 48 h. The cells were then stimulated for 6 h with poly (I:C). Protein expression levels of CRFB5 and P-TYK2 were detected by Western blotting (**D**), while the expression of antiviral genes (*PKR*, *MX1*, *ISG15*, and *IFN-I*) was detected by RT-qPCR (**E**). The data are presented as mean ± SD (*n* = 3). Significance is shown as *, *p* < 0.05; **, *p* < 0.01 in comparison to the controls.

Previous research has demonstrated that CRFB5 serves as a typical receptor chain for all type I IFNs in teleost fish and participates vitally in the antiviral immune response mediated by IFNs [5]. To further explore the response of CRFB5 to the poly (I:C)-induced immune response in silver carp, we studied the expression of CRFB5 and its mediated pathway following poly (I:C) stimulation. As illustrated in Figure 5A, upon poly (I:C) stimulation, *CRFB5* expression was significantly up-regulated in the spleen and HKCs of silver carp, and the protein levels of CRFB5 and P-TYK2 were also increased in HKC cells (Figure 5B). Furthermore, the overexpression of CRFB5 activated STAT1 and ISRE luciferase activities, with enhanced activation observed after poly (I:C) stimulation (Figure 5C). In addition, we selected a small interfering RNA (si-CRFB5-3) with a significant down-regulation function (Figure S2) to further verify the effect of CRFB5 on the JAK/STAT pathway. As shown in Figure 5D, transfection with si-CRFB5-3 significantly inhibited the expression of CRFB5 and P-TYK2 proteins. Meanwhile, the transcription levels of antiviral genes (*PKR*, *Mx1*, *ISG15*, and *IFN-I*) were significantly reduced (Figure 5E). Taken together, these findings indicate that CRFB5 is involved in poly (I:C)-induced antiviral responses through the JAK/STAT signaling pathway.

### 2.6. MiR-30b-5p Regulates the CRFB5-mediated JAK/STAT Signaling Pathway

**Figure 6:**
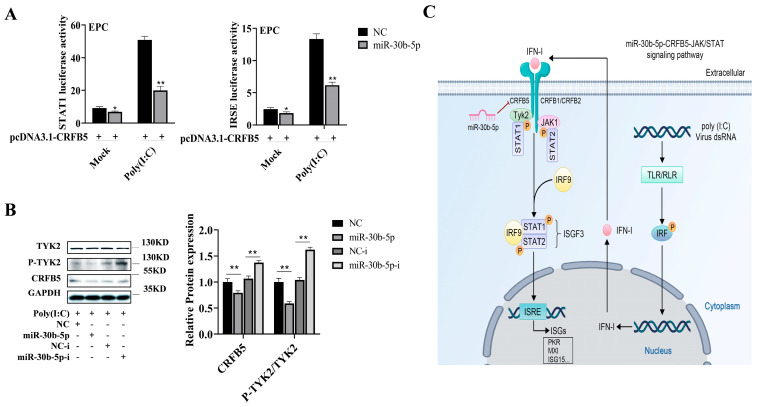
MiR-30b-5p-negative regulation of the CRFB5-mediated JAK/STAT signal pathway. (**A**) Following the co-transfection of EPC cells with the expression plasmid pCDNA3.1-CRFB5 and either the STAT1 or IRSE luciferin reporter plasmid, along with miR-30b-5p or NC at 48 h, cells were activated with poly (I:C) for 6 h, and luciferase activity was measured. (**B**) Silver carp HKCs were transfected with miR-30b-5p or NC and miR-30b-5p-i or NC-i for 48 h. Cells were then activated with poly (I:C) for 6 h, and Western blotting was performed to determine the levels of phosphorylated TYK2 (P-TYK2) and CRFB5 protein expression. (**C**) Model schematic of the regulatory role of miR-30b-5p in the JAK/STAT signaling pathway via targeting CRFB5. Data are presented as means ± SD (*n* = 3). Significance is shown as *, *p* < 0.05 **, *p* < 0.01 in comparison to the controls.

In view of the above findings, miR-30b-5p can regulate the expression of the antiviral genes, and miR-30b-5p can target CRFB5, which participates in the innate immunity induced by poly (I:C) through the JAK/STAT pathway. Thus, we looked into the possibility of a regulatory mechanism for miR-30b-5p on the CRFB5-mediated JAK/STAT signal pathway. As presented in Figure 6A, miR-30b-5p significantly suppressed the CRFB5-induced activation of STAT1 and ISRE, especially following poly (I:C) stimulation. Additionally, miR-30b-5p down-regulated the expression of CRFB5 and P-TYK2, while miR-30b-5p-i had the opposite effect (Figure 6B). These results unequivocally demonstrate that miR-30b-5p negatively regulates the CRFB5-mediated JAK/STAT signaling pathway in response to poly (I:C) stimulation (Figure 6C).

## 3. Discussion

As an integral part of the agricultural industry, aquaculture has emerged as a vital avenue for ensuring a high-protein diet for human consumption. However, with the continual expansion of aquaculture operations, the issue of disease in freshwater aquaculture has become increasingly pronounced. Among these diseases, viral infections stand out due to their rapid spread, broad impact, and the challenges they pose for prevention and control. These are the main diseases causing economic losses in aquaculture. Therefore, exploring the potential immunomodulatory mechanism of teleost fish holds significant importance for disease prevention and control. Numerous studies have demonstrated that microRNA is engaged in regulating innate immune response in teleost fish [20], but the reports in silver carp have not been studied in detail. In this study, poly (I:C) was used as an inducer to simulate the infection with a double-stranded RNA virus, revealing for the first time the regulatory role of miR-30b-5p in the immune response of silver carp via the CRFB5/JAK/STAT signaling pathway.

miRNA has a significant role in regulating viral infections. Viral infection triggers the activation of innate sensors within host cells, which recognize viral components and initiate a signaling cascade involved in mounting an antiviral response. Consequently, host miRNA expression is modulated to participate in the immune response. At present, there have been a large number of reports regarding miRNA participation in the antiviral response of teleost fish. For example, rhabdovirus-induced microRNA-210 targets STING/MITA to regulate the antiviral innate immune response in fish [21]. MiR-2188-5p promotes replication of the reovirus (GCRV) and inhibits the IFN-I response in grass carp (*Ctenopharyngodon idellus*) [22]. In our study, we discovered that miR-30b-5p in silver carp was significantly up-regulated when stimulated by poly (I:C). It was also proven that the miR-30b-5p of silver carp was involved in regulating the antiviral reaction induced by poly (I:C). Previous research has shown that the miR-30 family can regulate oxidative stress, apoptosis, autophagy, inflammation, and tumor progression in mammals [23,24,25]. With the development of the research, the changes in the miR-30 family’s immune response during environmental stress and the development of teleost fish have been reported [26]. Additionally, miR-30b-5p plays a broad regulatory role in Gram-negative bacterial infections in silver carp according to our earlier studies [27]. However, there are few reports on the antiviral effect of the miR-30 family in teleost fish, and its exact role in the innate immunity is still unknown to a great extent. The specific regulatory mechanism of miRNA still needs further investigations.

In order to combat viral infection, vertebrates release the cytokine interferon, which is crucial to the innate immune response. Previous research has demonstrated that teleost fish possess a mammalian-like IFN-I-mediated signal transduction pathway. As shown in Figure 6C, pattern recognition receptors, including RLR and TLR, induce and secrete IFN-I when host cells are stimulated by the dsRNA virus or its mimics. IFN-I binds to its receptor and induces the expression of antiviral genes through the conserved JAK-STAT signaling pathway to clear the virus in cells [28,29]. Fish IFN-I receptors belong to cytokine receptor family B (CRFB), among which all type I IFNs have the same receptor chain (CRFB5), and their involvement in the regulation of interferon antiviral response has been reported [5,7]. In our research, we found that the expression of type I interferon and CRFB5 in silver carp spleen tissues was up-regulated following poly (I:C) stimulation, which implied that CRFB5 might be involved in regulating the interferon antiviral response of silver carp. The antiviral response of teleost interferon mainly initiates the transcription of downstream ISG genes through the conserved STAT1 pathway, while the IFN-stimulated response element (ISRE element) is very conserved in vertebrate ISGs [30,31]. Consistent with the results of previous studies [7,32,33], STAT1 and the ISRE luciferase reporting system were utilized to prove that silver carp CRFB5 participated in antiviral reactions through the JAK/STAT signaling pathway in this study. Endogenous non-coding RNAs with known seed sequences selectively bind to the 3′-UTR region of target genes, thereby regulating their mRNA or protein-level expression [6].

This work showed that miR-30b-5p targets CRFB5 and participates in regulating the CRFB5 expression at the post-transcriptional level. The regulatory function of miRNAs is mainly mediated by target genes. For example, studies on host–virus interactions have demonstrated that miR-489 and microRNA-203 inhibit RIG-I signaling pathways in miiuy croaker by targeting TRAF6 and MDA5, respectively [34,35]. In this study, the target gene of miR-30b-5p was determined to be CRFB5 using luciferase reports and subsequent tests. Additionally, it was demonstrated that miR-30b-5p negatively regulates the JAK-STAT signaling pathway by targeting CRFB5. The results enrich the innate immune regulation network of miRNAs in teleost fish. The JAK/STAT signaling pathway is engaged in the regulation of numerous biological processes, including encouraging liver cancer cells to proliferate, migrate, and invade [36], ameliorating acute viral myocarditis [37], and inhibiting HBV infection [38]. However, over-activation of the JAK-STAT signaling pathway leads to an imbalance of host immune homeostasis and cause various immune system diseases [39,40,41]. In this study, we found that miRNA-30b-5p can mediate the JAK-STAT antiviral signaling pathway as a negative regulator, which avoids the over-activation of the JAK/STAT signaling pathway in silver carp and provides a new therapeutic measure for the host–pathogen immune response in teleost fish. The pivotal role of miRNA-mediated gene regulation has been established; however, the immunoregulatory role of miR-30b-5p in other teleost fish remains uncertain, and further research is needed to determine whether its antiviral immune response that targets CRFB5 is universal. And so far, the immune function of CRFB5 is unknown to a large extent in teleost fish. The miR-30b-p/CRFB5 axis is involved in the regulation of other immune pathways in silver carp, which still needs to be explored and studied. Furthermore, due to the complexity of the regulatory network of miRNA, according to different target genes, miRNA may have both positive and negative regulatory functions throughout the host–pathogen infection process. Therefore, further research on the function of the miRNA/mRNA regulatory axis in fish defense responses is required in the future.

In conclusion, our findings highlighted CRFB5 as a novel target of miR-30b-5p in silver carp and revealed for the first time that miR-30b-5p negatively regulates the JAK/STAT signaling pathway to mediate the antiviral immune response in silver carp via targeting CRFB5, thereby maintaining immune system stability. These results not only provide further theoretical groundwork for investigating how miRNAs act as negative feedback regulators in teleost fish antiviral immunity but also suggest that miRNAs may serve as potential therapeutic measures to prevent excessive immune response.

## 4. Materials and Methods

### 4.1. Inducing Infection in Silver Carp

Silver carp specimens, approximately 50 g in weight and in good health, were obtained from Jiutai Experimental Farms of the Fishery Sciences Academy of Jilin. Prior to experimentation, all fish were acclimatized to aerated aquariums at a temperature of 25 ± 2 °C for a duration of 2 weeks. Before the formal experiment, the tissues of silver carp (liver, spleen, and kidney) were randomly extracted for bacterial isolation and the cell infection experiment to ensure no other pathogenic infection was present. The silver carp were randomly divided into two groups, the poly (I:C) stimulation group (poly (I:C)) and the control check group (CK), each consisting of 20 fish (12 for experimental purposes, with the remaining utilized for supplementary purposes). Fish in the experimental group were administered intraperitoneal injections of 200 µL of poly (I:C) (2.5 mg/mL, InvivoGen, San Diego, CA, USA), while those in the CK group received equivalent injections of saline solution. Splenic tissue samples were collected from each group at various time points post injection (6, 12, and 24 h), rapidly frozen in liquid nitrogen, and stored at −80 °C for subsequent analysis. All procedures involving laboratory animals adhered to the JLAU regulations for animal experiments (JLAU08201409), and ethical approval was obtained from the Jilin Agricultural University Laboratory Animal Welfare and Ethics Committee.

### 4.2. Cell Culture and Cell Stimulation Experiment

The human embryonic kidney cell line (HEK-293T) of passage 8 obtained from BFB (BLUEFBIO, Shanghai, China) was cultured at 37 °C in DMEM (High Glucose, Hyclone, Logan, UT, USA) supplemented with 10% fetal bovine serum (FBS) and 1% penicillin–streptomycin solution (100×, Beyotime, Beijing, China) in a 5% CO_2_ incubator. Epithelioma Papulosum Cyprini (EPC) cells of passage 12 (preserved by the Fisheries Teaching and Research Office of Jilin Agricultural University, Changchun, China) were cultured at 26 °C in M199 medium (Hyclone, Logan, UT, USA) supplemented with 10% FBS and 1% penicillin–streptomycin solution in CO_2_ incubators. Macrophage cells (HKCs) were isolated and purified from silver carp head kidneys following the method described by Bi et al. [42] and were cultured at 26 °C in L-15 medium (Hyclone, Logan, UT, USA) supplemented with 15% FBS and 1% penicillin–streptomycin solution. For poly (I:C) stimulation experiments, cells were seeded in 6-well plates at a density of 4 × 10^7^ per well and allowed to incubate overnight. The next day, the cell culture medium was changed. Cells of the experimental group were replaced with fresh L-15 complete medium containing different final concentrations of poly (I:C) (1, 2.5, and 5 μg/mL, InvivoGen, San Diego, CA, USA) for stimulation, while the CK group cells were replaced with equal amounts of poly (I:C)-free L15 complete medium. Subsequently, cells were harvested at the specified poly (I:C) stimulated time points, 3, 6, and 12 h, for RNA extraction. Unstimulated cells were collected as controls. Three biological replicates were planned for each experiment.

### 4.3. Plasmid Construction and Transfection

To construct the full-length expression plasmid pcDNA3.1-CRFB5 for CRFB5, specific primers were designed to amplify the coding and 3′UTR region of silver carp CRFB5, which were then inserted into the pcDNA3.1-Flag tag vector (Invitrogen, Carlsbad, CA, USA) using Hind III and Xho I restriction sites. Similarly, the 3′UTR region of the CRFB5 gene was amplified with specific primer and cloned into the reporter vector pmir-GLO luciferase and mVenus-C1 green fluorescent protein (GFP) using Sac I and xho I, and Sac I and Sal I restriction sites, which was referred as WT-3′UTR and GFP-WT-3′UTR, respectively. Additionally, a mutant-CRFB5-3′UTR reporter plasmid (referred to as MT-3′UTR and named GFP-MT-3′UTR) was constructed using the Mut Express II Rapid Mutagenesis Kit V2 (Vazyme, Nanjing, China). Primer sequences are provided in Supplemental Table S1. Each constructed plasmid was validated by Sanger DNA sequencing and extracted using an endotoxin-free plasmid DNA Miniprep kit (TIANGEN, Beijing, China). Cell transfection experiments were conducted using Lipofectamine 3000™ (Invitrogen, Carlsbad, CA, USA) according to the manufacturer’s instructions.

### 4.4. Prediction of the miR-30b-5p Target Gene

To further elucidate the function of miR-30b-5p, two computational methods—Targetscan (v5.0) [43] and MiRanda (v3.3a) [44]—were employed to predict its target genes. According to the predicted efficacy of targeting, the predictions were arranged as calculated by utilizing the context and site scores (i.e., the threshold is Targetscan_Score ≥ 50; MiRanda_Energy < −10). Finally, the intersection of the two pieces of software was taken as the final target gene of the miR-30b-5p.

### 4.5. miRNA Mimics, Inhibitors, and RNA Interference

Mimics and inhibitors for miRNA-30b-5p, as well as siRNAs for CRFB5, were procured from GenePharma (Suzhou, China). Specific sequence information is shown in Table 1. Following the manufacturer’s instructions, mimics or inhibitors or siRNAs, along with their corresponding negative controls, were transfected into HKC cells using Lipofectaime 3000™ (Invitrogen, Carlsbad, CA, USA) to assess their accuracy via qPCR or Western blot analysis. Three biological replicates were planned for each experiment.

### 4.6. Dual Luciferase Reporter Gene Detection Assays

To identify miRNA target genes, reporter plasmids containing the CRFB5 3′UTR, either wild-type or mutant, along with the negative control (NC), or miR-30b-5p, or NC inhibitor (NC-i), or miR-30b-5p inhibitor (miR-30b-5p-i), were transfected into HEK-293T or EPC cells. Cells were harvested at various intervals, and the Dual Luciferase Reporter System (Promega, Madison, WI, USA) was utilized to assess cellular activity. Relative luciferase activity values were normalized using Renilla luciferase as an internal control. Three biological replicates were planned for each experiment.

### 4.7. qPCR Detection

Total RNA was extracted using Trizol (Sangon, Shanghai, China) following the manufacturer’s instructions. As previously described [27], the quality and concentration of RNA were assessed by employing a bioanalyzer (Agilent Bioanalyzer 2100, Pleasanton, CA, USA), and samples with RNA Intensity Number (RIN) values greater than 8 were selected for subsequent experiments. To evaluate the mRNA and mature miR-30b-5p expression, reverse transcription was used to convert RNA (unified 500 ng) to cDNA using the miRNA 1st-Strand cDNA Synthesis Kit (by stem-loop) (Vazyme, Nanjing, China) and the PrimeScript™ RT reagent Kit with gDNA Eraser (Takara, Tokyo, Japan), respectively. Real-time quantitative PCR detection was carried out on the LightCycler96 real-time PCR system (Roche, Basel, Switzerland) using the TB Green Premix Ex Taq™ II (Takara, Tokyo, Japan) for mRNA and the miRNA Universal SYBR qPCR Master Mix (Vazyme, Nanjing, China) for miR-30b-5p. GAPDH and U6 were used as internal reference genes for mRNA and miR-30b-5p detection, respectively. Primer sequences are provided in Supplemental Table S1. The specific reaction system and reaction procedure of mRNAs are as follows: The 20 μL reaction system included 2 × TB Green Premix Ex Taq (10 μL), cDNA (2 μL), upstream and downstream primers (10 μM, 0.4 μL each), and RNase-free ddH_2_O (7.2 μL). The initial denaturation was 30 s at 95 °C, a reaction using the two-step method for 40 cycles of 3 s at 95 °C, and then annealing for 30 s at 60 °C. The specific reaction system and reaction procedure of miRNA are as follows: The 20 μL reaction system included 2 × miRNA Universal SYBR qPCR Master Mix (10 μL), cDNA (1 μL), Specific Primer and mQ Primer R (10 μM, 0.4 μL each), and ddH_2_O (8.2 μL). The cycling conditions were as follows: initial denaturation for 5 min at 95 °C, followed by 40 cycles of two steps (10 s at 95 °C, and annealing for 30 s at 60 °C). The relative quantitative 2^-ΔΔCT^ threshold cycle (CT) method was employed to calculate the relative expression levels. Three biological replicates were planned for every experiment.

### 4.8. Western Blotting

Total cellular proteins were extracted using the RIPA lysis buffer (Beyotime, Beijing, China), and protein concentrations were determined using the BCA Protein Assay Kit (Beyotime, Beijing, China). Polyacrylamide gel electrophoresis (SDS-PAGE) was used to evaluate protein expression in accordance with established protocols. Various antibodies (Abs) were used for protein detection: Anti-Flag mAb at 1:1000 dilution (Beyotime, Beijing, China), anti-CRFB5 mAb at 1:500 dilution (ThermoFisher, Waltham, MA, USA), anti-TYK2 and P-TYK2 pAb at 1:1000 dilution (Abclonal, Woburn, MA, USA), and anti-GAPDH pAb at 1:1000 dilution (Abclonal, Woburn, MA, USA). HRP-conjugated anti-Mouse and anti-Rabbit IgG (Abbkine, Beijing, China) were used at 1:5000 dilution. Immunoreactive proteins were visualized using a BeyoECL Plus (Beyotime, Beijing, China) and imaged with an Amersham Imager 680. ImageJ (v1.53c) software was used to analyze the protein intensities. Three biological replicates were planned for each experiment.

### 4.9. Statistical Analysis of Data

Data were analyzed using the 2^−∆∆CT^ method, and inter-group comparisons were performed using one-way analysis of variance (ANOVA), followed by multiple comparison tests [45]. Results are presented as the mean ± standard deviation (SD), and differences between groups were determined using the *t*-test with statistical significance defined as *p*-values < 0.05.

## Figures and Tables

**Table 1:** The sequence information of mimics, inhibitors, and siRNAs.

Primer ID	Sequences (5′ to 3′)
miR-30b-5p (sense)	UGUAAACAUCCUACACUCAGCU
miR-30b-5p (antisense)	CUGAGUUAGGAUGUUUACAUU
NC (sense)	UUCUCCGAACGUGUCACGUTT
NC (antisense)	ACGUGACACGUUCGAGAATT
miR-30b-5p-i	AGCUGAGUUAGGAUGUUUACA
NC-i	CAGUACUUUUUGUGUAGUACAA
si-CRFB5-1 (sense)	CGGCUUUCAAUCCCGUAATT
si-CRFB5-1 (antisense)	UUACGGAUGAAACGCCGTT
si-CRFB5-2 (sense)	CCGACAUUUAUUACCUUAUTT
si-CRFB5-2 (antisense)	AUAAGUAAUCAUGUGGTT
si-CRFB5-3 (sense)	GCUGCUGUGUUUCUCAGTT
si-CRFB5-3 (antisense)	UUGUAGAAGUCAGCAGCTT
siRNA-NC (sense)	UUCUCCGAACGUGUCACGUTTTT
siRNA-NC (antisense)	ACGUGACACGUUCGGAGAATT

## Data Availability

All data are included in the results and Supplementary Data.

## Supplementary Materials

The following supporting information can be downloaded at https://www.mdpi.com/article/10.3390/ijms25115712/s1.
