# Supplementary material for: Poly (I:C)-Induced microRNA-30b-5p Negatively Regulates the JAK/STAT Signaling Pathway to Mediate the Antiviral Immune Response in Silver Carp (Hypophthalmichthys molitrix) via Targeting CRFB5
Source: Int J Mol Sci. 2024 May 24;25(11):5712. doi: 10.3390/ijms25115712 (PMC11172372; doi:10.3390/ijms25115712)
Supplement: Supplementary file 1 [file ijms-25-05712-s001.zip › ijms-2998368-supplementary.pdf]

Poly (I:C)-Induced miRNA-30b-5p Negatively Regulates the JAK/STAT Signaling Pathway to Mediate the Antiviral Immune Response in Silver Carp (*Hypophthalmichthys molitrix*) via Targeting CRFB5

Meng Liu†1, Huan Tang†1, Kun Gao[1], Xiqing Zhang[1], Zhenhua Ma[1], Yunna Jia[1], Zihan Yang[1], Muhammad Inam[1], Yunhang Gao*1, Guiqin Wang*1 and Xiaofeng Shan[1]

[1] Department of Veterinary Medicine, College of Animal Science and Technology, Jilin Agricultural University, Changchun 130118, China.

liumeng4610@163.com (M.L.), tanghuan202308@163.com, (H.T.), KunGao213@163.com (K.G.), Zhangxiqing1020@163.com (X.Z.), mazhenhua1030@163.com (Z.M), 15590074644@163.com (Y.J.), yyangzihan622@163.com (Z.Y.), dr.inam@sbbu.edu.pk (M.I)

* Corresponding authors: *E-mails:* gaoyunhang@163.com (Y. G), wgqjlau@aliyun.com (G.W)

† These authors contributed equally to this work.

Table S1. qRT-PCR primer sequence information in this study

Primer ID	Sequences (5' to 3')	bp
CRFB5-3'UTR-WT-F	GCGGAGCTCGTGCAGTTGACTTGTTCTTGTT	264
CRFB5-3'UTR-WT-R	TGCCTCGAGTCTGATGTGCTCCATGTTCTC	
CRFB5-3'-UTR -GFP-F	GCGGAGCTCGTGCAGTTGACTTGTTCTTGTT	264
CRFB5-3'-UTR -GFP-R	ACGCGTCGACTGCACAGTCGCTATTCTAACT	
CRFB5-3'UTR-MUT-F	GAGATGATcacccgtgTTAATATTTGCTTCTAGGAAGGATTTTT	264
CRFB5-3'UTR-MUT-R	AAcacgggtgATCATCTCAATGCAGAACTGAACATA	
CRFB5-CDS&UTR-F	CCCAAGCTTGGAACCGAAAGAAAGCTGAAAG	1812
CRFB5-CDS&UTR-R	TGCCTCGAGTCTGATGTGCTCCATGTTCTC	
qPCR-CRFB5-F	CTCCCGACATGTATTACCTTATCC	128
qPCR-CRFB5-R	CAGACAGCGTCACCACATTA	
qPCR-GAPDH-F	GGGATTGTCGCTCATCTATCTT	104
qPCR-GAPDH-R	GTTGCTGTACCCAAACTCATTG	
qPCR-IFN-I-F	GAGGACCAGGTGAAGTTTCTT	121
qPCR-IFN-I-R	CAGTCTGTAGGTTCCACTGC	
qPCR-PKR-F	ATCTGAGGATGCACGACTCTCATC	108
qPCR-PKR-R	GCTCGCACAAGGACTTATGGC	
qPCR-MX1-F	ACGCCTCACAGACTATGTGCC	103
qPCR-MX1-R	TCAGCAGTTTGACCACATCTGCC	
qPCR-ISG15-F	GGCACAACAAGAGATTGTGAAG	102
qPCR-ISG15-R	TGGCAAGCAGGATGGAAATA	
qPCR-miR-30b-5p	GCGCGTGTAAACATCCTACAC	/
qPCR-U6-F	CTCGCTTCGGCAGCACA	116
qPCR-U6-R	AACGCTTCACGAATTTGCGT	
Universal primer-R	AGTGCAGGGTCCGAGGTATT	/
RT-miR-30b-5p	GTCGTATCCAGTGCAGGGTCCGAGGTATTCGCACTGGATACGACAGCTGA	/

Table S2. The putative immune-related target genes of miR-30b-5p by programs TargetScan and miRanda.

miRNA	Predicted Target gene	TargetScan score	miranda Energy
miR-30b-5p	**IGKV4-1**	60	-18.39
	**Lurap1l**	83	-12.33
	**Bcl10**	92	-17.11
	**TNFSF13B**	67	-29.09
	**ufl1**	74	-13.39
	**MITF**	72	-10.61
	**ASMT**	74	-10.68
	**VTN**	75	-10.81
	**Traf4**	82	-14.1
	**CRFB5**	95	-14.9

Screening the immune-related target genes with TargetScan score≥50 and miRanda Energy <-10. Among them, CRFB5 had the highest score in the gene prediction program.

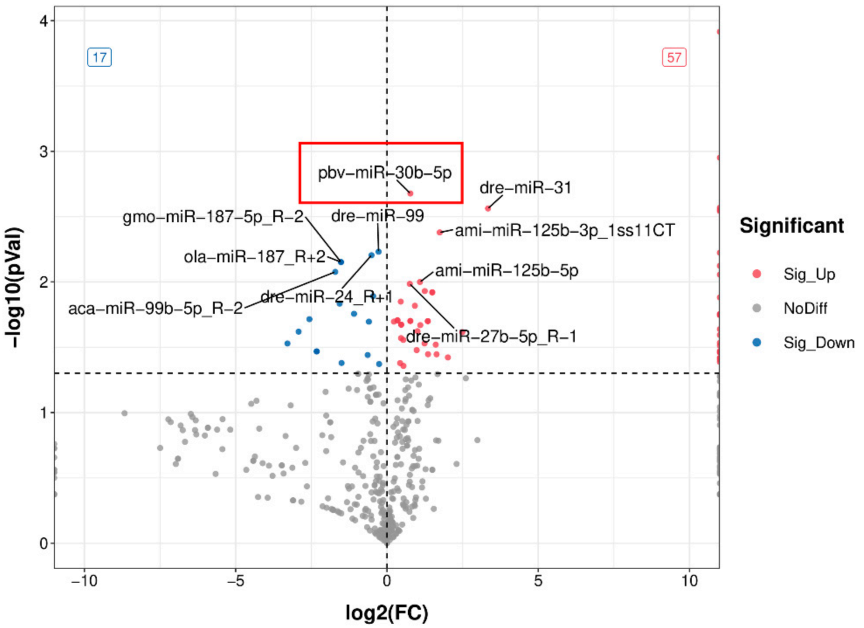

Figure S1. The volcano plot of DEmiRNAs in two challenge groups(CK vs. poly(I:C)). Red represents up-regulated significantly differentially expressed miRNAs, blue represents down-regulated significantly differentially expressed miRNAs, and gray dots represent non-significantly differentially expressed miRNAs; In red boxes are upregulated miRNA screened for this study

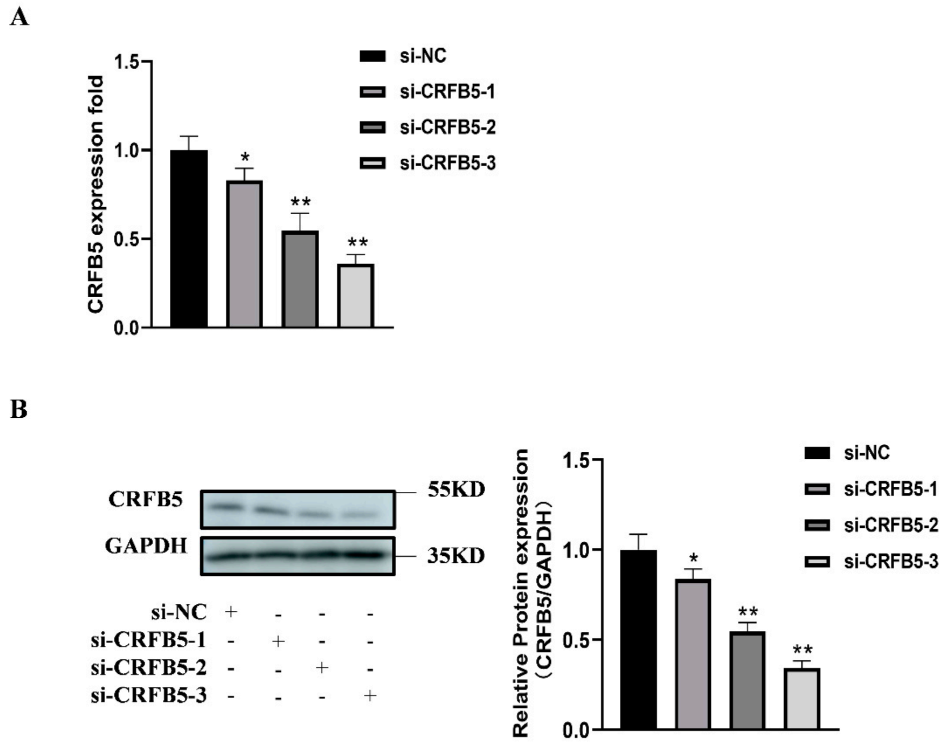

Figure S2. Relative mRNA and protein levels of CRFB5 in HKC cells. si- CRFB5-1, si- CRFB5-2, si-CRFB5-3 or siRNA- negative control (NC) by qPCR assays (A) and western blotting (B), respectively.
